# *N,N*-Dimethylformamide (DMF) Usage in Epoxy/Graphene Nanocomposites: Problems Associated with Reaggregation

**DOI:** 10.3390/polym9060193

**Published:** 2017-05-27

**Authors:** Jiacheng Wei, Mohd Shahneel Saharudin, Thuc Vo, Fawad Inam

**Affiliations:** Faculty of Engineering and Environment, Department of Mechanical and Construction Engineering, Northumbria University, Newcastle upon Tyne NE1 8ST, UK; jiacheng.wei@northumbria.ac.uk (J.W.); mohd.saharudin@northumbria.ac.uk (M.S.S.); thuc.vo@northumbria.ac.uk (T.V.)

**Keywords:** *N,N*-dimethylformamide (DMF), epoxy, graphene, reaggregation, nanocomposites

## Abstract

DMF is one the most commonly-used solvents for preparing graphene nanocomposites. Various processing variables for DMF are being used for the preparation of epoxy/graphene nanocomposites. Whilst the emphasis of all of these reported studies are on the improvements in mechanical, and other properties, of the epoxy/graphene nanocomposites, there is no study investigating how DMF affects the processing and how it is associated with the final properties of the nanocomposites. In this work, different dosages of DMF have been used to prepare nanocomposites. Mechanical testing, X-ray diffraction (XRD), dynamic mechanical analysis (DMA), thermogravimetric analysis (TGA), and scanning electron microscopy (SEM) have been used to analyze the effectiveness of DMF dosage on the properties of processed nanocomposites. Larger dosages of DMF are not always ideal for dispersing graphene as it promotes reaggregation of graphene during the processing.

## 1. Introduction

Since its discovery in 2004 [1], graphene has rapidly gained both academic and industrial interest because of its outstanding properties, such as high surface to volume ratio, high aspect ratio, extremely low electrical resistivity, high thermal conductivity, and high mechanical strength and modulus [2]. These fascinating properties have attracted extensive research in recent years with ever-increasing scientific and technological impetus.

Epoxy is a widely used thermoset material due to its superior mechanical properties, thermal stability, solvent resistance, and ease of processing [3]. Applications of epoxy and its nanocomposites include aerospace, automotive, marine, sports materials, construction, structures, electrical and electronic systems, biomedical devices, thermal management systems, adhesives, paints, coatings, industrial tooling, and other general consumer products [4]. Its versatile nature makes it a good candidate to replace many conventional materials, for instance, epoxy-based materials have replaced woods in the majority of boats and various sports goods.

For epoxy/graphene nanocomposites, graphene can significantly improve the physical and chemical properties of the matrix at extremely low loadings [5], and this enhancement could only be achieved when graphene is homogeneously dispersed in the matrix. The uniformly-dispersed graphene could share external stress to avoid stress concentration and impede crack propagation, which leads to the improvements in the mechanical properties. On the contrary, poorly-dispersed graphene acts as stress raiser and causes stress concentration, resulting in the deterioration of the mechanical properties [5].

The exploration of property enhancements of epoxy/graphene nanocomposites is rapidly advancing. However, in practical terms, graphene is not suitable to disperse in epoxy just by simple mixing, as graphene tends to reaggregate in the matrix due to the strong van der Waals force even after homogenisation. Dispersing them in polymer resin with relatively high viscosity is difficult. Therefore, using solvents as dispersing media has been widely accepted and regarded as the simplest method to distribute isolated graphene homogeneously in the nanocomposite materials.

For example, in some studies [6,7,8,9,10,11,12,13], graphene was dispersed in acetone and improvements in the final properties of the nanocomposites were reported with 1 g/L concentration of graphene. In some studies [14,15,16,17,18,19,20,21,22,23,24,25], graphene was dispersed in water, ethanol, tetrahydrofuran (THF), dichloromethane (DCM), and *N,N*-dimethylformamide (DMF) at 1 g/L, with remarkable improvement in the final properties. Dispersal of graphene in DMF, ethanol, and acetone have even been demonstrated at different concentrations, such as 1 g/2 L, 1 g/3 L, or 1 g/10 L [26,27,28,29,30,31,32,33,34,35]. For some other studies, solvents with unknown concentrations were used. For example, some reports [36,37] reported using DMF in the processing of epoxy/graphene nanocomposites and the final materials showed enhanced mechanical properties and resistance to fatigue crack growth. Some other articles [38,39,40] reported the usage of ethanol and the nanocomposites showed improved load transfer efficiency, as well as an improved glass transition temperature. Other solvents, like isopropanol [41,42], THF [43,44], butanone [45,46], acetone [47,48], and dichloromethane [49], have also been reported in the processing of epoxy/graphene nanocomposites. However, in all of these studies, the dosage of solvents had not been specified. Therefore, these analyses cannot be references for the usage of solvents in epoxy/graphene nanocomposites preparation. The dispersion effectiveness of popular solvents was reported by Shih et al. [50] using computational analyses of NMP = DMSO > DMF > GBL > water. However, there were no comments on the optimization of processing variables (e.g., dosage of solvent). To date, there has been no research publication addressing the effects of solvent volume on the properties of the epoxy/graphene nanocomposites. This study, therefore, examines the usage of DMF solvent for the processing of epoxy/graphene nanocomposites.

## 2. Materials and Methods

**Figure 1:**
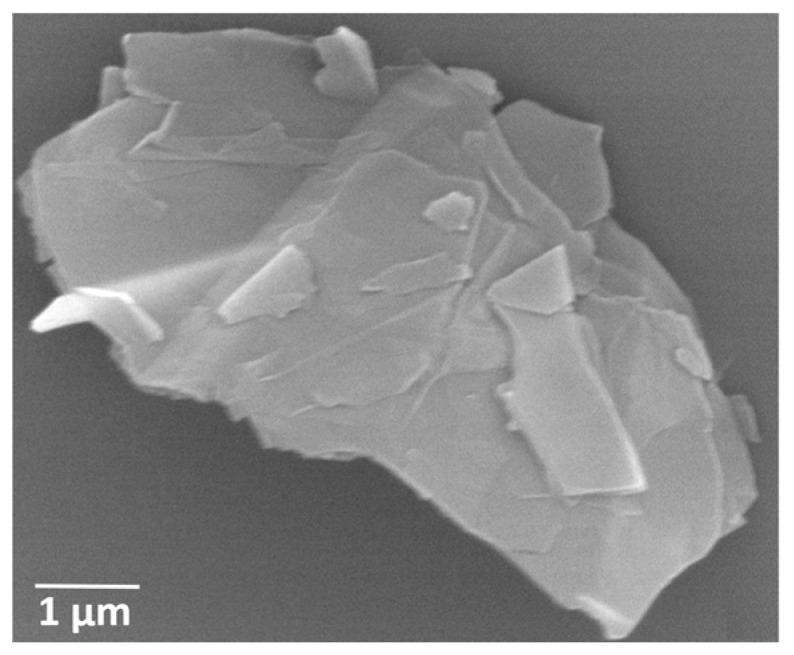
SEM image of a graphene nanoplatelet.

The epoxy matrix used in this study consists of EPOPHENTM EL5 bisphenol A based liquid epoxy (EP) and EPOPHENTM EHA57 diamine hardener (HD), purchased from Polyfibre UK Ltd. (Birmingham, UK) This epoxy system is a multi-purpose resin offering good all-round properties with the epoxy group content of 4.76–5.25 mol/kg. The viscosities of the liquid epoxy and hardener at room temperature are 12,000–15,000 and 45 cps, respectively. To prepare the epoxy material (EP + HD), 100 parts by weight of liquid epoxy were mixed to 50 parts by weight of hardener. Graphene nanoplates were purchased from Graphene Laboratories Inc. (Calverton, NY, USA) (product name: AO-3). The graphene nanoplatelets, according to the manufacture’s technical datasheet, have a specific surface area of 80 m^2^/g, the average lateral size and thickness are 4.5 μm and 12 nm, respectively. Figure 1 shows the SEM image of graphene nanoplatelets. DMF used in this work was purchased from Sigma-Aldrich (Sigma-Aldrich Company Ltd., Gillingham, UK) with a purity of 99.9%.

Five sets of nanocomposites filled with 0.3 wt % graphene were prepared. One set of samples was prepared as a reference using neat epoxy only, marked as G-0.3. Another four sets were prepared with different dosages of DMF. 

**Figure 2:**
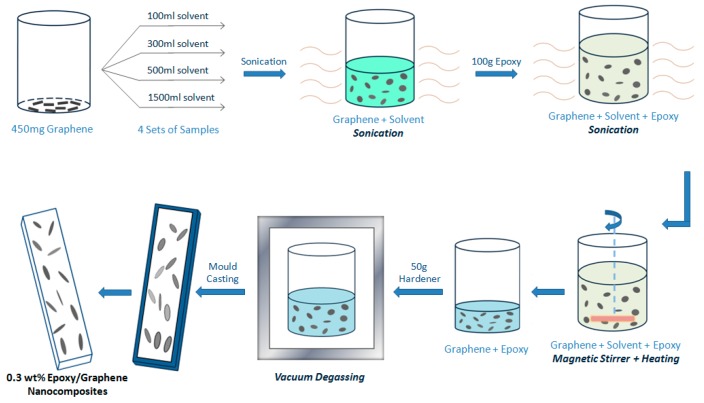
Schematic of the preparation of nanocomposites.

To understand the relationship among solvent dosage, graphene dispersion state, and the properties of the nanocomposites, 0.3 wt % epoxy/graphene nanocomposites were prepared by using different dosages of DMF. The dosages prepared were 100, 300, 500, and 1500 mL. Graphene (0.45 g) was first dispersed in a specified dosage of DMF (100, 300, 500, and 1500 mL, respectively, marked as D-100, D-300, D-500, and D-1500, accordingly) and bath sonicated for 0.5 h. Epoxy monomer was then added to the dispersion and sonicated for another 0.5 h. To remove the DMF solvent, the mixtures were heated to 150 °C with stirring. The mixture with 100 mL of DMF was only heated for 4 h, the mixtures with 300 and 500 mL DMF were heated for 8 h, and the mixture with 1500 mL DMF was heated for 16 h to evaporate the solvent. All mixtures were weighed to ensure full removal of DMF. The mixtures were then cooled to room temperature and the hardener was added with hand stirring for 5 min, followed by 5 min bath sonication. Vacuum degassing was then carried out to remove the entrapped air bubbles. Subsequently, the mixtures were mold-cast and cured at room temperature for 6 h, followed by 6 h post-curing at 80 °C. Figure 2 shows the schematics of the sample preparation.

Tensile, three-point bend, and fracture toughness tests were conducted with a universal testing machine (Instron 3382, Instron Corporation, Norwood, MA, USA), and the crosshead speed was kept at 2 mm/min for all tests. Tensile properties were measured according to ASTMD638 (Type V geometry). The three-point bend test was conducted according to ASTM D790 with specimen dimensions of 3 mm × 12.7 mm × 48 mm. A single-edge-notch three point bending (SEN-TPB) specimen was used to test mode-I fracture toughness (*K*_1C_) according to ASTM D5045. The specimen dimensions were 3 mm × 6 mm × 36 mm with a crack length of 3 mm. The notch was made at the middle of the sample and tapped to sharpen by a razor blade. The *K*_1C_ was calculated using Equation (1):

(1) $$K_{1C}=\;\frac{P_{max}f\left(\frac{a}{w}\right)}{BW^{1/2}}$$

where, *P_max_* is the maximum load of the load-displacement curve, *f(a/w)* is constant related to the sample geometry and was calculated using Equation (2), *B* is sample thickness (mm), *W* is sample width (mm), and *a* is crack length (kept between 0.45 and 0.55 *W*). The critical strain energy release rate (*G*_1C_) was evaluated using Equation (3) where *E* is the Young's modulus obtained from the tensile tests (MPa), and *v* is the Poisson's ratio of the polymer, taken to be 0.35.

(2) $$f\left(\frac{a}{w}\right)=\;\frac{\left[\left(2\;+\;\frac{a}{w}\right)\left\{0.0866\;+\;4.64\left(\frac{a}{w}\right)\;-\;13.32{\left(\frac{a}{w}\right)}^{2}\;+\;14.72{\left(\frac{a}{w}\right)}^{3}\;-\;5.6{\left(\frac{a}{w}\right)}^{4}\right\}\right]}{{\left(1\;-\;\frac{a}{w}\right)}^{3/2}}$$

(3) $$G_{1C}=\;\frac{{K_{1C}}^{2}\left(1\;-\;v^{2}\right)}{E}$$

A dynamic mechanical analyzer (DMA) (Model 8000, Perkin Elmer, Waltham, MA, USA) was used to determine the storage modulus (*E*’) and loss factor tan δ. All tests were conducted by using the temperature sweep method (temperature ramp from 30 to 150 °C at 5 °C/min) at a constant frequency of 1 Hz. Thermogravimetric analysis (TGA) of the nanocomposites was carried out with a TA Instruments Q500 thermal analyzer (TA Instruments, New Castle, DE, USA). The temperature range was from room temperature to 600 °C at a ramp rate of 5 °C/min under N_2_ atmosphere. The structure of the epoxy/graphene nanocomposites was examined using X-ray diffraction (XRD) carried out with a Siemens D-5000 diffractometer (Bruker Corporation, Coventry, UK) using a Cu Kα radiation source (λ = 0.15406 nm) with step size of 0.02°. Scanning electron microscopy (SEM) analysis was carried out using a FEI Quanta 200 microscope (FEI Corporation, Hillsboro, OR, USA) to examine the fracture surfaces of nanocomposites. The fractured surfaces were cut from the specimens and a layer of gold was applied using an Emscope sputter coater, model SC500A (Quorum Technologies Ltd., Laughton, UK). For tensile, flexural, fracture, and hardness tests, six specimens were tested for each set of conditions and mean values were then reported.

## 3. Results and Discussion

### 3.1. Mechanical Properties of Nanocomposites

**Figure 3:**
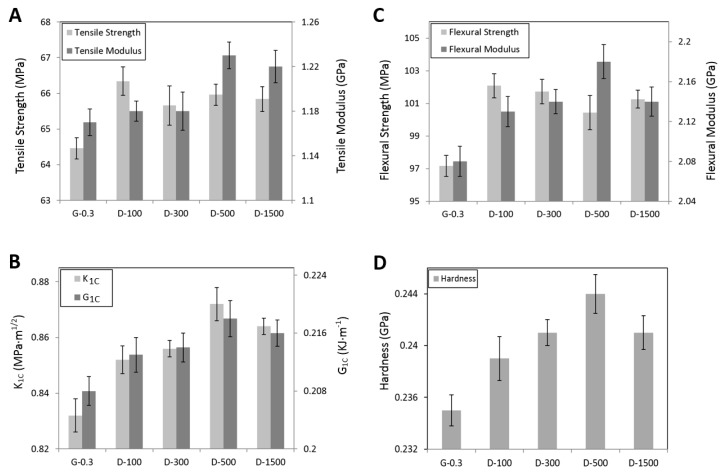
Mechanical properties of epoxy/graphene nanocomposites: (**A**) tensile properties; (**B**) flexural properties; (**C**) fracture properties; and (**D**) hardness.

Most work on epoxy/graphene nanocomposites aims at improving the mechanical properties of the nanocomposites, while the dispersion state of graphene, in return, affects the macroscopic properties of the matrix. Therefore, the mechanical properties of the nanocomposites have been tested and summarized in Figure 3.

As can be seen from the figure, G-0.3 shows the lowest mechanical properties. Overall, the mechanical properties of the nanocomposites showed enhancements when DMF was used. For tensile strength and flexural strength, D-100 samples showed the highest values, whereas for tensile the modulus, flexural modulus, *K*_1C_, *G*_1C_, and hardness, D-500 samples showed the highest values. The general improvements of these properties are due to good distribution of graphene by using DMF. Uniformly-dispersed graphene could improve the energy-absorbing capacity of the material, restrict the epoxy chain mobility, and shorten the distance among cross-linking points, thus increasing the properties of the nanocomposites [5]. 

However, when too much solvent was used, e.g., 1500 mL DMF in this work, lower properties were observed in comparison with the samples prepared with less solvent, e.g., 100 or 500 mL DMF. This decrease can be associated with the reaggregation of the graphene, which occurred due to the large dosage of DMF used. 

### 3.2. TGA Test of Nanocomposites

**Figure 4:**
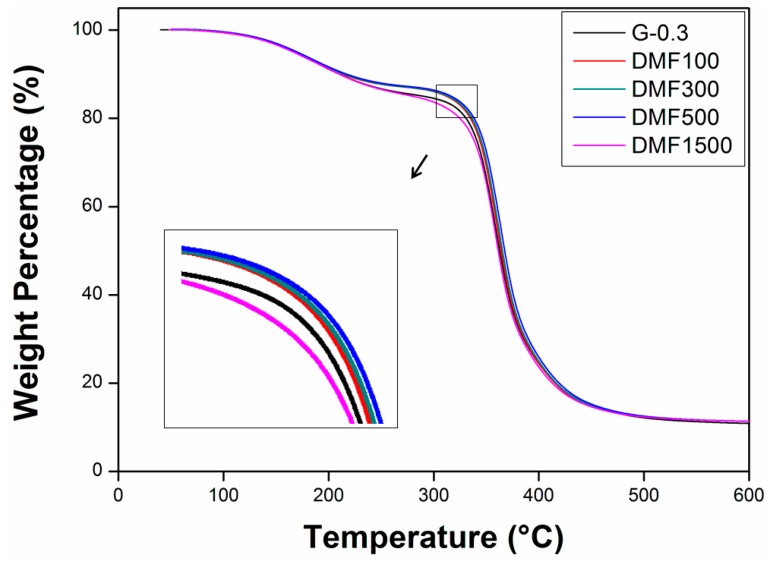
TGA curves of the nanocomposites.

Thermal decomposition is one of the fundamental thermal properties and is critical for practical applications. Figure 4 shows the TGA curves of the nanocomposites in a nitrogen atmosphere. It can be seen that all samples have a similar two-stage weight loss, indicating that they have a similar thermal degradation mechanism. The first weight loss from 100 to 230 °C was attributed to the decomposition of small molecules on the side chain. The second weight loss, occurring from 250 to 500 °C, shows the decomposition of the main polymer chain. 

As can be seen from the Figure 4, all D-100, D-300, and D-500 samples showed lower decomposition rates as compared to G-0.3 samples, which means D-100, D-300, and D-500 samples have higher thermal stabilities. The reason can be ascribed to the fact that graphene can increase the cross-linking density of the epoxy, and other thermoset polymers, as elaborated elsewhere [51,52,53], thus leading to higher thermal stability. In general, the increased dispersion of graphene in D-100, D-300, and D-500 samples resulted in a higher heat capacity of the nanocomposites. However, when too much solvent was used, e.g., 1500 mL DMF in this work, non-uniformly-dispersed graphene decreased the properties of nanocomposites; thus, D-1500 samples showed the highest decomposition rate under heating.

### 3.3 DMA Results of Nanocomposites

**Figure 5:**
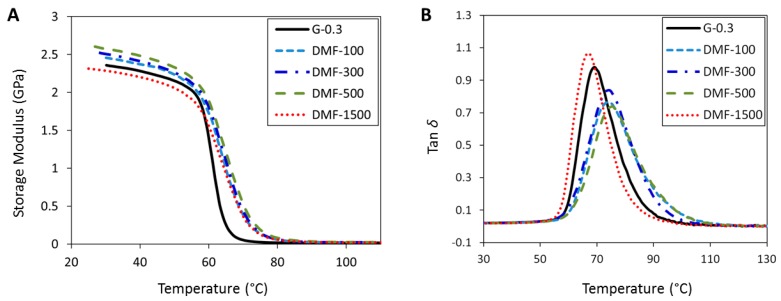
DMA results of nanocomposites: (**A**) storage modulus; and (**B**) tan δ.

Figure 5A shows the storage modulus (*E*’) as a function of temperature for epoxy/graphene nanocomposites. As can be seen from the figure, the storage modulus of G-0.3 is 2.35 GPa. With the increasing dosage of DMF, D-100, D-300, and D-500 samples show increased storage moduli of 2.45, 2.52, and 2.60 GPa respectively. However, with the further increase of the DMF dosage, D-1500 shows a decreased storage modulus with the value of 2.31 GPa. This is the lowest value among all of the samples.

The glass transition temperature (*T_g_*) characterizes the segmental motion of polymers and was taken as the temperature value at the peak of the tan *δ* curves, as shown in Figure 5B. In the figure it can be seen that the tan δ peak is observed at 69.28 °C for nanocomposites prepared with no DMF and, for nanocomposites prepared with 100, 300, and 500 mL DMF, *T_g_* shifted to a higher temperature. This can be ascribed to the fact that the uniformly-dispersed graphene restricted molecular mobility of the epoxy matrix, thus leading to the increased *T_g_* values. Among all of these increments, 500 mL DMF-prepared nanocomposites showed the highest *T_g_* of 75.57 °C, which is more than 6 °C higher than that of G-0.3 samples, while a 5 °C increment in *T_g_* was observed for D-100 and D-300 samples. The reason for this improvement can be explained by the effect of graphene on the cross-linking structure of the nanocomposites. Generally, the cross-linking density means the concentration of cross-linked bonds per volume. As for a typical polymer nanocomposite, the higher the cross-linking density, the stronger the polymer chains bond to each other, therefore leading to a higher *T_g_* of the nanocomposites. However, samples prepared with 1500 mL DMF show the lowest *T_g_* value of 65.78 °C. The likely reason for this decrease is that graphene, in a liquid matrix, tends to reaggregate over time, especially in a low viscosity medium. As a larger dosage of DMF requires a longer time for complete evaporation, the reaggregation of graphene is more likely to occur. Comparing with the structures of well-dispersed epoxy/graphene nanocomposites, the non-uniformly-dispersed graphene decreased the *T_g_*.

To summarize the mechanical properties, it has been described in the previous published research that due to the strong van der Waals force on dispersed graphene sheets, graphene tends to reaggregate in liquid matrix with the passage of time [54,55,56]. From the analysis (Figure 3, Figure 4 and Figure 5) it can, therefore, be concluded that large dosage of DMF (e.g., D-1500) provides an ideal low viscous medium for graphene to re-agglomerate. It is also a time-consuming task to evaporate DMF completely; therefore, prevention of re-agglomeration with time can only be accomplished if minimal, but appropriate, dosages of DMF are used (e.g., 100 or 500 mL), as evidenced in this work.

### 3.4 SEM Images of Nanocomposites

**Figure 6:**
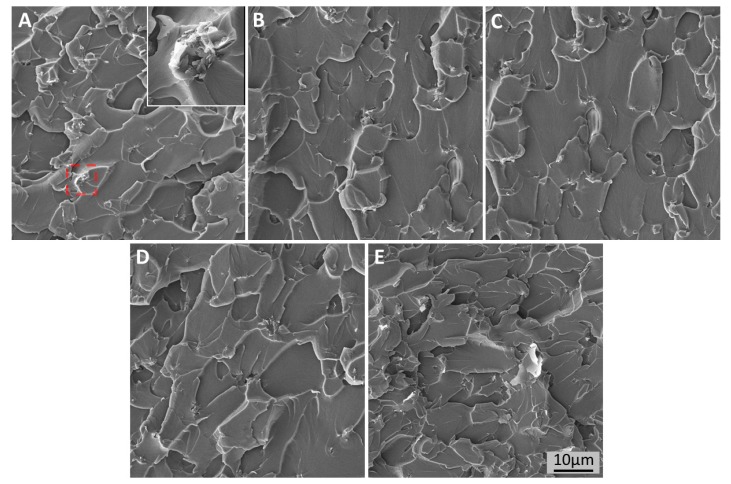
SEM images of fracture surfaces of (**A**) G-0.3; (**B**) D-100; (**C**) D-300; (**D**) D-500; and (**E**) D-1500.

The fracture surfaces of nanocomposites were examined by SEM and are shown in Figure 6. For G-0.3 samples, as shown in Figure 6A, some poorly-dispersed graphene can be seen on the surface. This poorly-dispersed surface features a poor interfacial interaction between the matrix and graphene. It also shows the brittle nature of the material and poor resistance to crack initiation and propagation. Compared with G-0.3, the fracture surfaces of D-100, D-300, and D-500 samples are relatively more uniform, as shown in Figure 6B–D. The clear fracture patterns show the sheet/sheet delamination as the fracture mechanism for the nanocomposites, and reveals that the usage of appropriate amounts of DMF are able to generate a uniform dispersion of graphene. The uniformly-dispersed graphene in the matrix can form a continuous network, which can release stress concentration. Additionally, uniformly-dispersed graphene could bridge growing cracks, thus stabilizing and stopping the cracks from developing into larger and harmful cracks. This also enhances the properties of nanomaterials. However, for D-1500 samples, as shown in Figure 6E, graphene aggregates could still be seen on the fracture surface. These aggregates form defects in the nanocomposites, act to concentrate the stresses locally, and cause localized weakness, thus causing large cracks and decreasing the properties of the nanocomposites.

### 3.5 XRD Results of Nanocomposites

**Figure 7:**
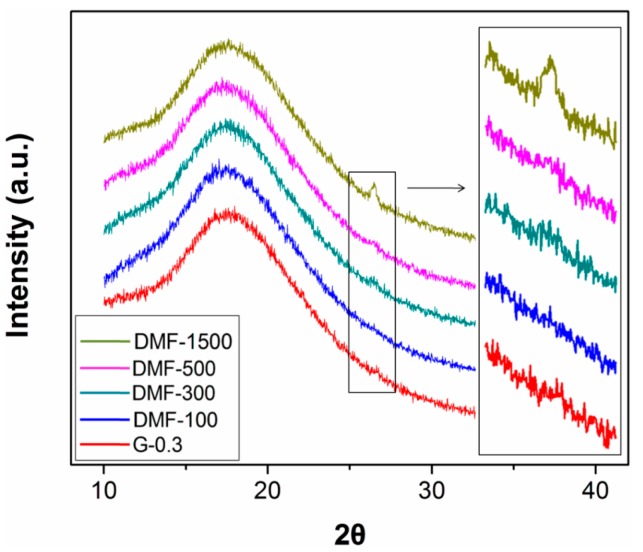
XRD patterns of nanocomposites.

Finally, XRD was used to characterize the structure of epoxy/graphene nanocomposites. As shown in Figure 7, all of the samples exhibit a wide diffraction from 11°–28°, which is caused by the scattering of the X-ray beam by cured epoxy molecules and shows the amorphous feature of the matrix. However, for samples prepared with 1500 mL DMF, there is a sharp shoulder peak of 2θ at 26.5°, which features the structure of graphite. 

This graphitic structure could only be caused by the agglomeration of graphene during processing. This result clearly shows that the use of a large dosage of DMF has induced reaggregation of graphene, which leads to the decrements in the properties.

In summary, polar solvents, like DMF, are responsible for providing a low-viscosity medium, which is an ideal environment for fullerenes to agglomerate [53]. It is also widely understood that bonding (either physical or chemical) can be accelerated by increasing the temperature. While drying, higher temperatures are responsible for promoting agglomeration between graphene sheets. This results in the triggering of van der Waals forces on graphene surfaces in close proximity with each other, leading to agglomeration. In practical terms, larger dosages of solvent require longer times for complete evaporation. Therefore, it can be further concluded that the long processing time, higher temperatures, and low-viscosity solvents are responsible for the promotion of reagglomeration of graphene. This has also been confirmed by other publications [54,55,56] as well. For example, in one of our previous works [56], reaggreation of graphene (with the passage of time) in various liquids was confirmed via UV–VIS spectroscopy. 

## 4. Conclusions

DMF was used to investigate the effects of solvent dosage on the preparation and properties of epoxy/graphene nanocomposites. This research provides guidelines for the usage of DMF solvents in the preparation of epoxy/graphene nanocomposites, and could also be a reference for other polymer composites where the use of solvents is required in the processing. Mechanical properties, TGA, DMA, SEM, and XRD were tested in this work. The results show that large dosage of solvents are responsible for decreasing the final properties of the nanocomposites. The long processing time, higher temperatures, and low viscosity of solvents are responsible for the promotion of the reagglomeration of graphene. These findings will have profound implications in nanocomposites manufacturing, as large amounts of solvents could be avoided from economic and health and safety perspectives. The processing time could be shortened causing less environmental pollution by reducing the amount evaporated solvents. These results help in optimisation and are having positive implications on the practical processing technology of nanocomposites. However, although the relationship between solvent dosage and the consequent processing of epoxy/graphene nanocomposites has been demonstrated here for the first time, it has not given the critical value for the best condition of dispersibility and processability. Therefore, more work needs to be conducted to fully understand the best usage of solvents.
